# Two Sporadic Cases of Legionellosis Associated with the Use of Domestic Ultrasonic Humidifiers

**DOI:** 10.3390/microorganisms12112139

**Published:** 2024-10-25

**Authors:** Javier Reinares Ortiz, Jorge Pérez-Serrano, Juana María González-Rubio, Fernando González-Camacho

**Affiliations:** 1 Public Health Center, 28806 Alcalá de Henares, Spain; reinaresoj@madrid.es; 2 Department of Environmental Health, Madrid-Salud, 28007 Madrid, Spain; 3 Department of Biomedicine and Biotechnology, School of Pharmacy, University of Alcalá de Henares, 28871 Madrid, Spain; jorge.perez@uah.es; 4 Legionella Reference Laboratory, National Centre for Microbiology, Instituto de Salud Carlos III, 28220 Majadahonda, Spain; jmgonzalez@isciii.es

**Keywords:** legionnaires’ disease, humidifier, sporadic case, *Legionella pneumophila*

## Abstract

Two sporadic cases of legionellosis occurring in consecutive years were confirmed by positive antigenuria to *Legionella pneumophila* serogroup 1 in individuals with limited mobility who were confined to their homes. Both cases had a history of using ultrasonic humidifiers and of low exposure to other possible sources of infection. This study was conducted through an expanded epidemiological survey and home inspection. Samples were collected from domestic hot water and humidifiers. Environmental isolates were characterised by immunoagglutination and immunofluorescence. The Dresden panel is employed for the determination of groups and subgroups of serogroup 1. The amoebae were isolated by water filtration and subsequent cultivation of the filters. Identification of the isolates was conducted through the sequencing of PCR products. In both cases, epidemiological studies identified the ultrasonic humidifiers as the probable source of infection. The presence of *Legionella pneumophila* was confirmed in the sanitary water installation and in the humidifiers, where high values of *Legionella pneumophila* serogroup 1 counts were also obtained. The isolates were characterised as Olda Oxford and Olda OLDA. Furthermore, in addition to the Legionella isolates, the presence of *Vermamoeba vermiformis* was also identified in samples obtained from domestic hot water and humidifiers in one of the residential addresses under investigation. It has been demonstrated that humidifiers can act as an amplification mechanism for pathogenic microorganisms when they are not cleaned and maintained correctly. This can pose a health danger, especially to people with previous respiratory pathologies, the immunosuppressed, and the elderly. Therefore, it is of the utmost importance that professionals who recommend the therapeutic use of this equipment should issue warnings regarding the importance of its correct use, cleaning, and disinfection. Finally, humidifiers should be considered in epidemiological surveys and in the study of sporadic cases.

## 1. Introduction

*Legionella pneumophila* (*Lpne*) is an environmental bacterium with the capacity to colonise the human-made water supply network, including cooling towers, building water systems, hot tubs, ornamental or recreational fountains, and others. From this point, the bacteria can reach the lungs through the inhalation of contaminated aerosols produced by the aforementioned devices [1,2,3]. *Legionella* spp. are the causative agent of legionellosis. This disease can manifest in two clinical forms. The most serious of these forms is Legionnaires’ disease (LD), which is characterised by severe pneumonia that has the potential to be fatal.

The genus *Legionella* comprises more than 70 species, with approximately half of these having been associated with human disease. Nevertheless, it is *Lpne* that is responsible for more than 90% of LD cases. A total of 16 different serogroups of *Lpne* have been identified. This classification is based on the use of monoclonal antibodies in the Dresden panel [4]; however, the agent most commonly associated with LD is SG1, which accounts for approximately 85% of reported cases [5].

The disease has a higher incidence in older individuals and in those with immunosuppression, with the potential for a fatal outcome in the absence of prompt and appropriate treatment at the onset of symptoms [6,7]. Furthermore, the incidence is related to the virulence of the *Lpne* strain, associating the Pontiac group with a high probability of causing LD, and the Olda group—being the most frequent isolate found in environmental sources—which is rarely associated with LD [7,8].

This disease can manifest as sporadic cases or as outbreaks and can occur at both the nosocomial and community level (75% of cases) or in association with travel [5]. In the majority of cases of sporadic legionellosis, the source of the infection remains unidentified. It is probable that the domestic environment represents a significant source of infection. However, research into these home environments is not conducted on a routine basis. It is therefore possible that a significant proportion of unidentified cases may be related to the individual’s place of residence. Among the potential sources of infection in the home environment, the primary sampling points are the endpoints of sanitary hot water (SHW), taps, and shower heads. It is noteworthy that other potential locations of interest include household equipment that contains a water reservoir with the capacity to produce aerosols, where the bacteria could undergo amplification and spread. Among these, domestic humidifiers may represent a significant source of infection. The increasing use of these devices in domestic settings, particularly among the elderly and individuals with respiratory ailments, coupled with the dearth of awareness regarding their potential microbiological hazards, underscores the necessity for a heightened focus on the associated health risks of these devices.

In this study, we present two cases of legionellosis where epidemiological and microbiological data suggest that an ultrasonic humidifier may have been the source of infection.

## 2. Materials and Methods

### 2.1. Epidemiological and Environmental Study

A comprehensive epidemiological investigation was conducted to ascertain the potential sources of infection in high-risk facilities and equipment at all potential acquisition sites (workplace, travel, hospital, community, and home) [9].

To conduct an environmental investigation, water samples were collected from the shower and tap in the bathroom of the private residence. Furthermore, samples were also obtained from the shower and faucet in the hospital room of the patient who was admitted. The presence of *Legionella* was determined in these samples through the collection of 1 L of water in containers containing thiosulfate. Moreover, the water within the personal humidifier tank was also sampled. In all instances, biofilm was extracted using a swab that was introduced into the water sample. Subsequent analysis was conducted through plate counting following membrane filtration, in accordance with the Spanish normative ISO in Water Quality [10].

### 2.2. Microbiological Characterisation of the Isolates

The isolates were cultivated in buffered charcoal yeast extract agar (BCYE) and BCYE without cysteine at 36 °C for a period of two days. Then, the isolates were identified via latex immunoagglutination (Legionella Latex Test Kit, Oxoid, UK). The confirmation of *Lpne* serogroup 1 (SG1) was achieved through immunofluorescence using polyclonal antibodies, while the group and subgroup of SG1 were identified through the utilisation of the complete Dresden panel of monoclonal antibodies [4].

### 2.3. Isolation and Characterisation of Protozoa

Each water sample was composed of a 500-millilitre aliquot of the sample collected from the showers and taps. The water was filtered through a 3.0 μm nitrocellulose membrane using the 3.0 μm Microfilter filtration equipment (ALBET) and the Microfiltration system (MILLIPORE, Burlington, MA, USA). Subsequently, the filtration membrane was placed on non-nutritive agar plates containing dead *Escherichia coli* dispersed on the surface to isolate the amoebae present in the samples. The plates were sealed with Parafilm^®^ (Amcor Flexibles North America, Oshkosh, WI, USA) to prevent water evaporation and incubated at 32 °C. The plates were observed under the microscope daily until trophozoites or cysts were observed. The amoebae were maintained at this temperature through repeated subcultures on non-nutritive agar plates until a clone (a strain derived from a single cyst or trophozoite) was isolated. The clone was then transferred to Peptone Yeast Glucose (PYG) liquid axenic medium in a 25 cm² flask at 32 °C. Trophozoite cultures were maintained in subcultures by transferring 500 μL of medium containing trophozoites and cysts to fresh medium on a weekly basis [11]. The clone was identified by DNA extraction and PCR employing the universal eukaryotic primers 1492R and 528F [12] and the *Acanthamoeba*-specific primers JDP1 and JDP2. The PCR products were sequenced (Research Support Unit in Medicine and Biology, University of Alcalá de Henares). Finally, the sequences were analysed using the Basic Local Alignment Search Tool (BLAST) (National Library of Medicine), using as reference the sequences from the GeneBank, EMBL, and DDBJ databases.

## 3. Results

### 3.1. Case 1

An 87-year-old woman with a history of asthma and limited mobility. The patient initially presented general symptoms including cough and general malaise. Ten days later, the patient’s condition had deteriorated, with the onset of stuporous behaviour and bilateral pneumonia, which led to her admission to the Internal Medicine Hospital. Following a positive result on the urine antigen test for *Lpne* SG1, the patient commenced treatment with levofloxacin, with favourable progress, and she was discharged from the hospital twelve days later.

Due to her limited mobility, the patient remained at home throughout the incubation period. During this time, the patient did not use the shower, instead washing herself at the sink in the only bathroom in the house. During the environmental investigation, the following facilities were identified as posing a risk: the SHW without a return circuit and the personal humidifier used by the patient. The sink faucet was of the single-lever variety. The concentration of free residual chlorine (FRC) in the cold water was 0.3 parts per million (ppm). The temperature of the hot water after one minute was recorded at 46.2 °C. The faucet diffuser filter was observed to contain a considerable quantity of retained debris. The humidifier had been used on an occasional basis for several months without any cleaning or maintenance. The humidifier was of the domestic ultrasonic variety, equipped with a 1.5-litre tank. The device was filled from the sink tap and was activated without first emptying the residual water from the previous use. A volume of 5 mL of water that remained from the previous use could be recovered from the tank during sampling.

In the hot water sample, 8 × 10⁴ CFU/L of *Lpne* SG1 were obtained, and in the humidifier sample, 3.8 × 10⁵ CFU/L of *Lpne* SG1 were counted belonging to the Olda Oxford subgroup. The detection of amoebae was negative in both samples.

### 3.2. Case 2

A 35-year-old male patient undergoing treatment for multiple sclerosis. During the incubation period, he remained confined, initially in the hospital, and subsequently at his home. The symptoms that the patient initially presented with were high fever, general malaise, and a dry cough. Despite four days of symptomatic treatment, the patient’s condition did not improve, prompting a visit to the emergency department of the referral hospital. There, he was diagnosed with left bilobular pneumonia. He was subsequently referred to the Pulmonology Department, where he commenced treatment with levofloxacin. Following an improvement in his condition, he was discharged from the hospital 13 days later.

**Figure 1:**
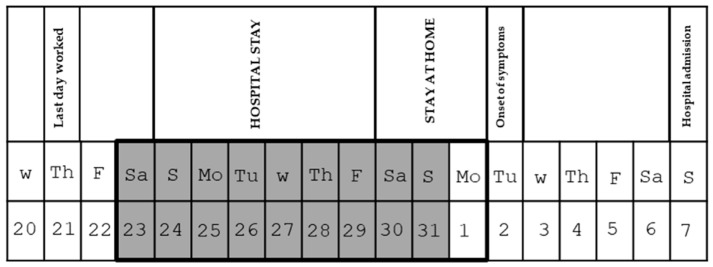
The chronology of Case 2 patient’s activity. The incubation period is framed in black, and the epidemiological and environmental research window is shaded in grey.

Nine days prior to the onset of symptoms, the patient was admitted to the Neurology Service, where he underwent scheduled treatment for his underlying disease with prednisone, acyclovir, and alemtuzumab (immunomodulator). During his hospitalisation, the patient did not require any respiratory equipment or access to the Emergency Department, although he did require the use of the hospital room’s bathroom. Subsequent to the administration of therapy, the patient remained confined to his place of residence. The patient’s timeline is illustrated in Figure 1.

The risk installations identified in his private home were the SHW system with a return circuit in addition to a personal domestic humidifier. In the hospital, the SHW system with a return circuit was identified as a potential risk. The shower in the home was observed to have a single-lever tap with a head that was in a satisfactory condition. The residual disinfectant level of the cold water was determined to be 0.6 ppm FRC. The temperature of the hot water was recorded as 45.8 °C after one minute. The faucet diffuser filter exhibited evidence of retained debris. Samples of 1 L and swab scrapings were obtained for the purpose of determining the presence of *Legionella* and amoebae. In the period preceding the onset of symptoms, the patient utilised a domestic ultrasonic humidifier. The device was equipped with a 4.2 L tank and a maximum nebulisation power of 230 mL/h. The tank was loaded at the shower faucet and was not generally emptied following each use. A volume of 1.5 L of water, representing the remaining capacity of the tank following its final use, was successfully recovered. The cleaning and maintenance conditions of the humidifier were suboptimal, and a red biofilm was observed on the surface of the tank, closing cap, and transducer.

The hot water sample from the home yielded 1.4 × 10⁴ CFU/L of *Lpne* SG1 Olda OLDA, while the humidifier sample returned 2.2 × 10⁵ CFU/L of *Lpne* SG1 Olda OLDA, along with the protozoan *Vermamoeba vermiformis*. This amoeba was present in both the home hot water sample and the humidifier sample. Samples obtained from the hot and cold water outlets of the shower and tap in the hospital room were found to be negative for *Legionella* spp., with a concentration of less than 1 CFU/L.

## 4. Discussion

This study presents two sporadic cases of community-acquired pneumonia related to the use of domestic ultrasonic humidifiers as the most probable source of *Legionella* infection.

Tap water has never been considered sterile; rather, the objective of disinfection is to reduce pathogenic microbes to levels that are deemed acceptable or tolerable. *Legionella pneumophila* is one of the most prevalent opportunistic pathogens in drinking water systems. Furthermore, other microorganisms, including several free-living amoebae such as *Acanthamoeba* species, can be found in these systems [13]. These amoebae are essential for the life cycle of *Legionella* [14,15]. The primary approach to managing opportunistic microorganisms in drinking water supplies is to control their growth. However, under certain conditions, these bacteria can proliferate to levels that may present a potential risk to public health. A previous epidemiological study has been published which evaluated the risk of transmitting legionellosis and the routes of exposure in a private home. In this study, the authors demonstrated that ultrasonic and cold mist humidifiers are the equipment that produce the highest exposure doses followed by the shower and the tap [16]. Other studies have proved that humidifiers can serve as a source of microbial emission [17,18,19] and that the water reservoir containing the device can act as a risk factor for infection by facilitating the growth of these microorganisms and amplifying the risk of infection [20]. Also, several documented cases of legionellosis in infants resulting from the domestic use of an ultrasonic humidifier have been reported [21,22,23].

In this study, the concentrations of *Legionella* in the humidifiers studied were found to be higher than in the tap samples analysed from the private home, with counts exceeding 10^5^ CFU/L in both cases. The observed increase in the Legionella concentration in the humidifiers, in comparison to the SHW counts, can be attributed to several factors. Primarily, the residual disinfectant present in the sanitary water used to refill tank humidifiers is gradually eliminated from these devices over time. Secondly, the water in the humidifier tank remains at room temperature, which provides an optimal environment for microbial proliferation. Moreover, the humidifiers were utilised by patients without daily removal of the water contained in the tank. Finally, inadequate cleaning conditions permit the growth and maturation of biofilms, which facilitate the colonisation and subsequent amplification of *Legionella* by protozoa. In case 1, the *Legionella pneunophila* strain identified in the humidifier was of serotype Olda Oxford, which is commonly found in the environment, but it is infrequently responsible for causing legionellosis [8]. In case 2, the *Legionella pneunophila* strain identified in the faucet diffuser filter and in the hot water sample was of serotype Olda OLDA, which is also found in the environment, and it is reported to be responsible for legionellosis in patients with immunosuppression [7].

The use of humidifiers is common practice in the homes of individuals with an elevated risk of developing of Legionnaires’ disease, such as the elderly or patients with respiratory issues. However, these devices are often not properly maintained and could be the source of community-acquired pneumonia by *Legionella*. Therefore, it is mandatory to include these devices in epidemiological surveys and investigations into sporadic cases of legionellosis in the community.

The heightened risk associated with this equipment, in comparison to the risk posed by domestic hot water systems, can be attributed to three key factors. Firstly, there is a notable increase in aerosolisation due to the capacity of these devices to generate respirable droplets. Secondly, there is an elevated level of exposure to aerosols due to the extended operating periods of these devices by patients. Furthermore, the duration of exposure to aerosols is prolonged, since the elevated relative humidity of the air could facilitate their persistence in the atmosphere without evaporation. Additionally, the concentration of *Legionella* in the reservoir is increased due to the combined effects of device inactivity, inadequate maintenance, and conducive conditions for bacterial proliferation.

The main limitation of this study is the absence of clinical samples to enable the identification and confirmation of the source of infection. However, the limited exposure to other potential sources of infection in these patients, the high levels of *Lpne* SG1 found in the reservoir water of the humidifiers, and the presence of amoebae (in one of the devices) suggest that the origin of these two sporadic cases is likely due to the inhalation of contaminated aerosols produced by the patients’ humidifiers.

## 5. Conclusions

The risk of transmission of Legionnaires’ disease through the misuse of humidifiers has been described in this work, especially when adequate cleaning and maintenance of the devices are not carried out. The risk of suffering from legionellosis in susceptible people is elevated; thus, it is essential that health professionals advise patients who utilise this equipment on the necessity for proper maintenance. Moreover, manufacturers must incorporate instructions on how to perform these tasks correctly. Ultimately, epidemiologists should consider these devices when investigating sporadic cases of community-associated pneumonia.

## Data Availability

The original contributions presented in the study are included in the article, further inquiries can be directed to the corresponding author.

## References

[1] Orkis L.T., Harrison L.H., Mertz K.J., Brooks M.M., Bibby K.J., Stout J.E. (2018). Environmental sources of community-acquired legionnaires’ disease: A review. Int. J. Hyg. Environ. Health.

[2] Collins S., Stevenson D., Bennett A., Walker J. (2017). Occurrence of Legionella in UK household showers. Int. J. Hyg. Environ. Health.

[3] Centers for Disease Control and Prevention (CDC) Controlling Legionella in Cooling Towers. https://www.cdc.gov/control-legionella/php/toolkit/cooling-towers-module.html.

[4] Helbig J.H., Bernander S., Castellani Pastoris M., Etienne J., Gaia V., Lauwers S., Lindsay D., Lück P.C., Marques T., Mentula S. (2002). Pan-European study on culture-proven Legionnaires’ disease: Distribution of Legionella pneumophila serogroups and monoclonal subgroups. Eur. J. Clin. Microbiol. Infect. Dis. Off. Publ. Eur. Soc. Clin. Microbiol..

[5] (2023). Legionnaires’ Disease—Annual Epidemiological Report for 2022.

[6] Dagan A., Epstein D., Mahagneh A., Nashashibi J., Geffen Y., Neuberger A., Miller A. (2021). Community-acquired versus nosocomial Legionella pneumonia: Factors associated with Legionella-related mortality. Eur. J. Clin. Microbiol. Infect. Dis. Off. Publ. Eur. Soc. Clin. Microbiol..

[7] de Miguel-Balsa E., Jaimez Navarro E., Cascajero A., González-Camacho F., González-Rubio J.M. (2024). Fulminant septic shock due to community-acquired pneumonia caused by Legionella pneumophila SG1 Olda OLDA ST1. Case report. J. Infect. Public Health.

[8] González-Rubio J.M., Cascajero A., Baladrón B., González-Camacho F. (2024). Characterisation of Legionella Clinical Isolates in Spain from 2012 to 2022. Microorganisms.

[9] Lee J.V., Joseph C. (2002). Guidelines for investigating single cases of Legionnaires’ disease. Commun. Dis. Public Health.

[10] (2017). Water Quality—Enumeration of Legionella.

[11] Axelsson-Olsson D., Olofsson J., Ellström P., Waldenström J., Olsen B. (2009). A simple method for long-term storage of Acanthamoeba species. Parasitol. Res..

[12] Edgcomb V.P., Kysela D.T., Teske A., de Vera Gomez A., Sogin M.L. (2002). Benthic eukaryotic diversity in the Guaymas Basin hydrothermal vent environment. Proc. Natl. Acad. Sci. USA.

[13] LeChevallier M.W., Prosser T., Stevens M. (2024). Opportunistic Pathogens in Drinking Water Distribution Systems—A Review. Microorganisms.

[14] Richards A.M., Von Dwingelo J.E., Price C.T., Abu Kwaik Y. (2013). Cellular microbiology and molecular ecology of Legionella-amoeba interaction. Virulence.

[15] Price C.T.D., Hanford H.E., Al-Quadan T., Santic M., Shin C.J., Da’as M.S.J., Abu Kwaik Y. (2024). Amoebae as training grounds for microbial pathogens. mBio.

[16] Hines S.A., Chappie D.J., Lordo R.A., Miller B.D., Janke R.J., Lindquist H.A., Fox K.R., Ernst H.S., Taft S.C. (2014). Assessment of relative potential for Legionella species or surrogates inhalation exposure from common water uses. Water Res..

[17] Dietrich A.M., Yao W., Gallagher D.L. (2022). Exposure at the indoor water-air interface: Fill water constituents and the consequent air emissions from ultrasonic humidifiers: A systematic review. Indoor Air.

[18] Hull N.M., Reens A.L., Robertson C.E., Stanish L.F., Harris J.K., Stevens M.J., Frank D.N., Kotter C., Pace N.R. (2015). Molecular analysis of single room humidifier bacteriology. Water Res..

[19] Tyndall R.L., Lehman E.S., Bowman E.K., Milton D.K., Barbaree J.M. (1995). Home Humidifiers as a Potential Source of Exposure to Microbial Pathogens, Endotoxins, and Allergens. Indoor Air.

[20] Lee J.H., Ahn K.H., Yu I.J. (2012). Outbreak of bioaerosols with continuous use of humidifier in apartment room. Toxicol. Res..

[21] Rebecca Mitting V.R., Grossman T., Whittaker E., Chalker V., Lai S., Hoffman P., Atkin S., Qureshi S., Hatcher J. (2020). Severe neonatal legionellosis associated with use of home humidifiers—A case report. Clin. Infect. Pract..

[22] West P.T., Brooks E.F., Costales C., Moreno A., Jensen T.D., Budvytiene I., Khan A., Pham T.H.M., Schwenk H.T., Bhatt A.S. (2022). Near-fatal Legionella pneumonia in a neonate linked to home humidifier by metagenomic next generation sequencing. Med.

[23] Bonilla Escobar B.A., Montero Rubio J.C., Martínez Juárez G. (2014). Legionella pneumophila pneumonia associated with the use of a home humidifier in an immunocompetent girl. Med. Clin..

